# Palliative radiotherapy and the introduction of a Rapid Access Palliative Clinic in a national radiation oncology network

**DOI:** 10.1007/s11845-023-03494-4

**Published:** 2023-08-22

**Authors:** Cian O’Leary, Sinead Cleary, Hannah Linane, Barbara Hamilton, Michelle Jennings, Yvonne Lee, Naomi Lavan, Maeve O’Reilly, Marie Twomey

**Affiliations:** grid.477842.a 0000 0004 0617 8547 St. Luke’s Hospital Rathgar, Highfield Road, Rathgar, Dublin 6, Ireland

**Keywords:** Mortality, Palliative, Radiotherapy, Rapid access palliative clinic

## Abstract

**Background:**

Palliative radiotherapy (PRT) is commonly used to treat symptoms of advanced cancer. PRT has been associated with elevated 30-day mortality (30DM). A Rapid Access Palliative Clinic (RAPC) can streamline the treatment process for patients receiving treatment.

**Aims:**

We reviewed the PRT practices in a radiation oncology network in Ireland, and the implementation of a RAPC. Patient outcomes were assessed to inform future treatment decisions.

**Methods:**

A retrospective review of all patients who received PRT over 6 months in 2018 in St. Luke’s Radiation Oncology Network (SLRON) was undertaken. We assessed 30DM rates, demographics and referral to specialist palliative care (SPC) services. Subsequently, a retrospective analysis was conducted of a RAPC which ran for 6 months from 2019 to 2020. We assessed treatment data and mortality.

**Results:**

Over 6 months, 645 patients commenced PRT in the SLRON. The 30DM for this cohort was 15.8% (*n* = 102), with most patients having lung primaries. Of the 30DM cohort, only 55% (*n* = 56) were referred to SPC services and only 26.4% (*n* = 27) had performance status recorded. Over 6 months, 40 patients attended 28 RAPCs. Of these, 88% (*n* = 35) received PRT. Single fraction therapy was utilised in 60% and 48% of patients underwent CT simulation and treatment on the same day. Ultimately, 75% of patients received SPC referral.

**Conclusions:**

Referral rates to SPC services and documentation of performance status were low in our 30DM retrospective review cohort. The RAPC facilitated quick treatment turnaround, fewer hospital visits and referral to SPC services.

## Introduction

Palliative radiotherapy (PRT) is a widely prescribed treatment modality for patients with metastatic or locally advanced non-curable cancer. Radiotherapy can be an effective treatment to alleviate pain from bony metastases [1]. It can also be used to palliate other symptoms from locally advanced primary tumours or metastatic deposits such as bleeding, dysphagia and neurological symptoms from brain metastases or malignant spinal cord compression [2]. Studies have found that 40% of patients with advanced cancer receive PRT, and 40–50% of radiotherapy treatments are delivered with palliative intent [3, 4].

PRT should be tailored towards the individual patient as it can also be associated with a significant treatment burden which can in turn have a negative impact on patients’ quality of life [5]. It can cause side effects which largely relate to irradiation of normal tissue within the treatment field, e.g. diarrhoea, when treating the lumbar spine and oesophagitis when treating mediastinal disease. PRT is only available in specialised cancer hospitals so patients may be required to travel long distances and attend multiple hospital visits for consultation, planning scans and treatment.

The aim of PRT should be to deliver a minimum effective dose over the shortest period of time to achieve symptom control. Patients who are likely to benefit should be selected appropriately. In particular, patients with short prognoses should be considered for hypofractionated treatment, or even omission of treatment. Meanwhile, patients with longer prognoses, e.g. > 1 year, who are at risk of recurrence of symptoms, may benefit from longer courses of radiotherapy [6, 7].

Performance status and prognosis are important factors to consider when individualising treatment. Patients with short prognoses may not live long enough to benefit from the treatment. There are various predictive models of survival available to aid prognostication, although these remain underutilised. Performance status (ECOG or KPS) is a recognised indicator of outcome in the prescription of PRT and is perhaps the most widely used tool for discerning suitability for treatment [8].

Prognostication is challenging and often inaccurate. Doctors are accurate (within 33% of actual survival) in a mere 20% of cases, being overly optimistic in the majority (63%) and overly pessimistic in 17% of cases [9]. Several studies have assessed prognostication tools, most notably the Chow and TEACHH models [10, 11]. Both utilise performance status and other individual patient factors (e.g. site of primary cancer and site of metastatic spread) to estimate prognosis. Utilising these tools helps identify patients at the extremes of the prognostic spectrum and better informs on suitability for treatment.

In the UK’s National Health Service (NHS), 30-day mortality (30DM) has been proposed as a marker for avoidable harm in those receiving PRT [12]. Studies in both the UK and US have demonstrated 30DM rates of between 12 and 24% in patients receiving PRT [13, 14]. A study by Gripp [15] revealed that amongst patients receiving PRT within the last month of life, one half had worsening symptoms despite treatment with palliative intent and one quarter died whilst on treatment. Factors associated with increased 30DM in PRT patients include male sex, older age, primary cancer (e.g. lung primaries), site irradiated, poor performance status and site of metastatic disease [12, 13, 16].

Patients who are referred for PRT usually have symptoms that warrant referral to specialist palliative care (SPC). These include pain from bony metastases, symptomatic brain metastases, dysphagia and bleeding [17]. Unfortunately, referral rates are often low, even in advanced disease. In one US study, patients who died within 30 days of radiotherapy were less likely to have SPC involved in their care compared to those who lived longer than 30 days (44% vs 71%) [14].

Palliative care aims to improve the quality of life of patients with serious or life-limiting illnesses through the management of their physical, psychosocial and spiritual issues [18, 19]. SPC services are those whose primary function is the provision of palliative and end-of-life care to patients [18]. The World Health Organisation (WHO) estimates that 40 million people worldwide each year require palliative care [19]. Involvement of SPC services early in a patient’s disease trajectory has been shown to improve quality of life and mood and assists with ethical decision-making for patients and their treating teams [20]. Early SPC referral can also help reduce aggressive treatments at the end of life [21].

The introduction of a Rapid Access Palliative Clinic (RAPC) can reduce the time from referral to radiotherapy services to initial consultation and treatment, thereby minimising hospital attendances for patients with advanced disease [22, 23]. A RAPC could also hypothetically improve referral rates to SPC for this patient cohort.

## Objectives

The objectives of this retrospective study were threefold.

- To review PRT practices, 30DM rates and SPC referrals in a large radiotherapy network in Ireland over a 6-month period
- To review the implementation of a dedicated RAPC over a subsequent 6-month period
- To utilise this information to inform future practices related to PRT and running of the RAPC to enhance patient care and quality of life

## Methods

### Retrospective review of referrals for PRT

Data from all patients who received PRT in the three sites of St. Luke’s Radiation Oncology Network (SLRON), Dublin, over a 6-month period (January 2018 to June 2018) was collected retrospectively from our electronic patient record (EPR) known as ARIA. Ethical approval was sought and obtained through the St Luke’s Hospital Research and Ethics committee.

Data was inputted into the Excel software, on a password-protected computer, on the hospital site. Information on patient demographics was collected (patient age, gender, primary diagnosis and treatment site). The time interval from the last session of radiotherapy to the date of death was calculated, to a maximum of 2 years (up until the point when data collection occurred). To address missing data from the EPR, additional information was obtained by liaising with the patients’ general practitioners and by referencing online public death records resources (www.RIP.ie). Patients were deemed lost to follow up if no status of/date of death could be determined from these sources.

The 30-day mortality (30DM) rates were calculated. 30DM refers to the proportion of patients dying within 30 days of receiving their last given fraction of PRT. For each patient in the 30DM cohort, referral to a SPC team in either the hospital or community setting was recorded. Documentation on ARIA regarding patient performance status and cancer staging in the 30DM cohort was also assessed.

### Review of Rapid Access Palliative Clinic

We carried out a retrospective analysis of patients who attended the RAPC between September 2019 and March 2020.

Patients were considered unsuitable to attend the RAPC if they met any of the criteria in Table 1, as these factors would bring additional complexity to the radiotherapy planning process.

**Table 1:** Exclusion criteria

1. > 3 sites of bone pain
2. Re-irradiation to a previously treated site
3. More complex planning techniques (3DCRT, IMRT)
4. Electronic medical device in situ and details not available

Clinics ran twice a week and had capacity for a maximum of 3 patients in each clinic. One of three slots was reserved for a same day turnaround case, with priority being given to an emergency treatment or patients coming from a distance > 50 km.

Data was collected on patient age, sex, diagnosis, date of referral, date of consultation, treatment site, radiotherapy dose and radiotherapy fractionation from the EPR. The rate of referral to SPC during their radiotherapy treatment, date of referral, date of treatment and dates of patients’ deaths was obtained from the EPR or public records.

## Statistical analysis

Data was entered into Excel. This software was used to carry out quantitative analysis on demographic information (mean, median, mode, standard deviation). Time frame analysis was carried out to determine 30DM and time from referral to RAPC to treatment.

## Results

### Results of PRT practices

In total, 645 patients received PRT in the 6-month period. Date of death was not available for 12.4% of patients (*n* = 80). At 2 years post completion of their PRT, 11.6% of patients (*n* = 76) were alive.

The 30DM rate for the group was 15.8% (*n* = 102). Of these patients, 55% (*n* = 56) were referred to SPC services. When grouped according to the primary cancer site, the largest proportion (35%, *n* = 36) had a diagnosis of lung cancer and the most common reason for referral was for whole brain radiotherapy (WBRT) for brain metastases (26.5%, *n* = 27). Additionally, 17.6% had bony metastases irradiated (*n* = 18). Table 2 includes characteristics of the 30DM group.

**Table 2:** Characteristics from the 30DM cohort (*n* = 102)

Characteristic	
Sex	
Male	55% (*n* = 56)
Female	45% (*n* = 46)
Median age (years)	66 (range 39–91)
Median time to death (days)	13 (range 0–30)
Primary cancer site	No. of patients
Lung	35.3% (*n* = 36)
Genitourinary	15.7% (*n* = 16)
Gastrointestinal	13.7% (*n* = 14)
Breast	12.8% (*n* = 13)
Melanoma	4.9% (*n* = 5)
Head and neck	4.9% (*n* = 5)
Sarcoma	3.9% (*n* = 4)
Lymphoma	2.9% (*n* = 3)
Uterine	2.9% (*n* = 3)
Primary brain	2% (*n* = 2)
Primary unspecified	1% (*n* = 1)
Reason for PRT	
Brain metastases/whole brain radiotherapy (WBRT)	26.5% (*n* = 27)
Pain	19.6% (*n* = 20)
Symptom control	14.7% (*n* = 15)
Spinal cord compression	12.8% (*n* = 13)
No record	8.8% (*n* = 9)
Misc	8.8% (*n* = 9)
Respiratory	4.9% (*n* = 5)
Slow progression	3.9% (*n* = 4)

Only 26.4% (*n* = 27) of patients in the 30DM group had a performance status recorded. Of these, the median ECOG was 2, and a third (*n* = 9) had an ECOG of ≥ 3. Only 49% (*n* = 50) of patients in the 30DM cohort had the staging of their malignancy recorded on ARIA. Where recorded, there was significant variation in terminology used.

Of note, 25.4% (*n* = 26) of the 30DM group died within a week of their last fraction of radiotherapy, with 2 patients dying on the same day as their last fraction. Of these 26 patients, only 14 were referred to SPC services.

### Results of review of Rapid Access Palliative Clinic

Between Sept 2019 and March 2020, 40 patients, representing 13 different primary tumour sites, attended 28 RAPCs. Following initial consultation, 93% (*n* = 37) of patients seen were booked for treatment. Radiotherapy was not recommended to the remaining 7% (*n* = 3) of patients as they did not have a targetable lesion to account for their symptoms. A further 5% (*n* = 2) of patients became too unwell to commence treatment (WBRT for brain metastases) and died within 6 and 8 days, respectively. In total, 88% (*n* = 35) patients were treated.

The largest proportion of patients (31%, *n* = 11) had a genitourinary primary, followed by lung (17%, *n* = 6), breast (14%, *n* = 5) and gastrointestinal primary (11%, *n* = 4). Five patients received radiotherapy to 2 sites and 1 patient received radiotherapy to 3 sites. The majority of treatments (83%, *n* = 35) were directed at painful bony lesions, followed by nodal metastases (7%, *n* = 3), brain (5%, *n* = 2), lung (2%, *n* = 1) and soft tissue disease (2%, *n* = 1).

**Fig. 1:**
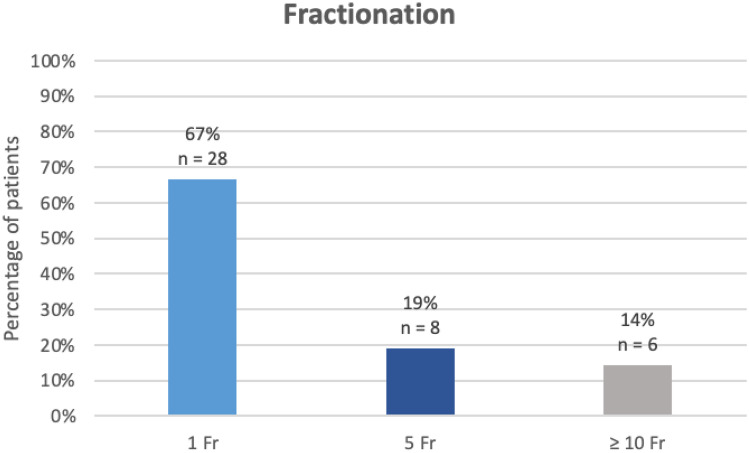
Fractionation schedules

The majority of treatments (67%, *n* = 28) were carried out in a single fraction, 19% (*n* = 8) were carried out in 5 fractions and the remaining 14% (*n* = 6) were carried out in ≥ 10 fractions (Fig. 1). When we looked at bone treatments alone, 80% (*n* = 28) were carried out in a single fraction. Data pertaining to time intervals at different stages of the patients’ care path is shown in Table 3.

**Table 3:** Time intervals

**Time interval**	**Median Time**	**Patients**	**IQR**
Referral to consultation	7 days	*n* = 33	4–8 days
Consultation to CT sim	51 min	*n* = 34	32–76 min
Consultation to starting RT	2 days	*n* = 35	0–6 days
Referral to starting RT	9 days	*n* = 33	7–13 days
Consultation to starting RT (SDT cases)	247 min	*n* = 17	229–380 min

*SDT* same day turnaround

Prior to the implementation of this clinic, initial consultation and treatment on the same day were reserved for emergency treatments and very occasionally patients with poor performance status. The RAPC saw these rates increase, allowing almost half of patients (49%, *n* = 17) to commence treatment on the same day as their initial consultation.

Half of patients (50%, *n* = 20) referred to the RAPC were already known to a community palliative care team. Of those who were not the 50% (*n* = 10) were referred via the RAPC.

**Fig. 2:**
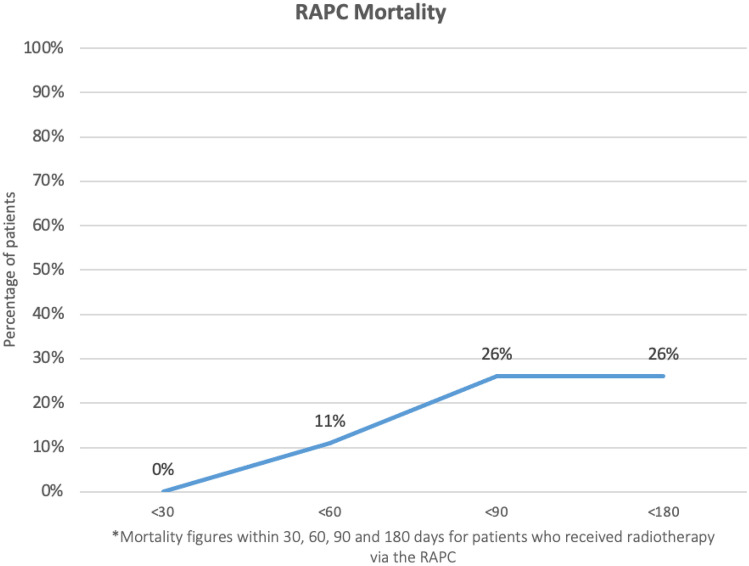
RAPC mortality

Time from the initial consultation to death was recorded if death occurred within 6 months. 30DM in this cohort was 0% (*n* = 35). A total of 11% (*n* = 4) died within 60 days, 26% (*n* = 9) died within 90 days and there were no additional deaths within 180 days. No patients were lost to follow-up (Fig. 2).

## Discussion

Whilst PRT can be an effective treatment, not everyone benefits. The 30DM rate from our retrospective review cohort was substantial and 4% (*n* = 26/645) of patients died within a week of their last fraction of RT. This is in keeping with similar studies of this nature [13, 14]. Risk factors associated with greater 30DM (older age, lung primary and ECOG ≥ 2) were prevalent in the cohort [12, 13, 16].

Referral rates to SPC services amongst patients who died within 30 days of their final radiotherapy session were poor, with 45% of patients in this cohort not referred for SPC. Given that early SPC referral has been shown to reduce aggressive therapies at the end of life [21], this represents a shortcoming in care for a vulnerable group of patients likely to benefit from SPC and a significant area for improvement in our delivery of their care.

Overall documentation of performance status, which is an important selection criterion for PRT [8], was poor in the 30DM cohort (26.4%, *n* = 27). This raises the concern that performance status is not always taken into consideration when it comes to selecting appropriate patients for PRT. We feel this represents a significant area for improvement, both in terms of documentation for future audit and reduction in risk of treatment burden for our patients. In addition, incorporating predictive models of survival into the RAPC could highlight patients with short life expectancies and prompt radiation oncologists to engage in discussions about advance care planning and referral to SPC services. Optimising pain and symptom management without radiotherapy may, in some instances, be more appropriate.

The 30DM rate amongst our RAPC patients was zero. This difference between the retrospective review group and the RAPC group may reflect differences in patient population and selection to proceed to PRT. The RAPC identified two patients who did not proceed to have treatment due to rapid clinical deterioration. In addition, the patients attending RAPC were receiving outpatient treatment and required a level of function and medical stability that would enable them to travel to the hospital to attend. The retrospective review group included inpatients in SLRON who, by virtue of their need to be cared for in an inpatient setting, may have been more symptomatic with higher care needs and a lower performance status.

Our data from the retrospective review demonstrated that not only was primary lung malignancy the most common diagnosis but that most patients in the 30DM cohort also had a primary lung malignancy. The most common reason for referral was for irradiation of brain metastases. Lung primaries have been demonstrated to confer an increased 30DM risk in PRT patients [13, 14, 16]. The Quality of Life after Treatment for Brain Metastases (QUARTZ) study shows that in patients with brain metastases from a non-small cell lung cancer (NSCLC) primary, best supportive care with dexamethasone is non-inferior to palliative WBRT and dexamethasone in terms of quality of life and is associated with similar survival outcomes [24]. These patients represent an at-risk group for whom treatment omission and best supportive care may be more appropriate. A RAPC could provide a useful outlet for triaging this cohort and referring directly to SPC services.

The majority of PRT treatments in our RAPC review were to painful bony metastases. These patients are good candidates for a RAPC which can facilitate initial consultation, planning scans and treatment delivery in a single visit. There is substantial evidence showing that single-fraction radiotherapy is equally as efficacious as multi-fraction radiotherapy for these lesions which again reduces the burden of hospital visits for patients [6].

## Limitations

We have noted several limitations in our data. Over 12% of patients in our retrospective review lack data on date of death. There are several factors which may have likely resulted in this, including lack of up-to-date correspondence from GPs, incomplete data logging on the ARIA system or cultural factors impacting reporting to RIP.ie. It does reflect a substantial gap in data, and we acknowledge the impact this has in accurate 30DM assessment for this cohort.

Overall, numbers referred to the RAPC were low and baseline characteristics, for example, primary tumour site and site irradiated, differ significantly between the two cohorts, meaning the two groups are not directly comparable. We suspect that patients who were treated via the RAPC were more likely to have shorter and single fraction treatment schedules compared to the retrospective review group; however, we do not have information on fractionation schedules in the retrospective review group.

## Conclusion

Our data has highlighted a significant number of patients who received PRT but may not have lived long enough to gain symptomatic relief from it. This emphasises two areas for improvement when it comes to optimising patient care:

1. The need for greater prudence in selecting patients for PRT via the consistent use of validated tools to assess prognosis accurately, and improved documentation of these assessments
2. The need for more frequent SPC referral in PRT patients with elevated 30DM risk

The intention to treat is always made in good faith, but as physicians we must aim to avoid treatment burden in our patients who have short prognoses. Medical therapies such as chemotherapy and cardiopulmonary resuscitation may often be considered futile and be withheld in the palliative setting, as the harm associated with them may outweigh the benefit [25, 26]; however, the same cannot be said for the sixth of the patients in our retrospective review who received radiotherapy in the last month of life. We propose an increased use of prognostication tools and patient performance status to aid physicians in identifying which patients will benefit from PRT and importantly, which patients will not. Performance status assessment was underutilised, representing the potential for avoidable harm in this at risk group.

PRT is, of course, just one aspect of holistic palliative care. Many patients would also benefit from the input of a SPC team who can optimise analgesia and recommend other adjuncts for symptom control [18–20]. Our retrospective review suggests SPC services may have been underutilised in our 30DM cohort, representing an area for improvement in the care experiences of our patients at the end of life.

The implementation of a RAPC allowed patients who required PRT to benefit from an efficient service by fast tracking initial appointments and reducing hospital visits. Additionally, the RAPC facilitated referral to SPC services. This is a new systematic approach for our service to facilitate access to treatment for palliative patients and ensure appropriate care is implemented.
